# Prevalence of patellofemoral pain and knee pain in the general population of Chinese young adults: a community-based questionnaire survey

**DOI:** 10.1186/s12891-018-2083-x

**Published:** 2018-05-24

**Authors:** Xingquan Xu, Chen Yao, Rui Wu, Wenjin Yan, Yao Yao, Kai Song, Qing Jiang, Dongquan Shi

**Affiliations:** 1 Department of Sports Medicine and Adult Reconstructive Surgery, Drum Tower Hospital, School of Medicine, Nanjing University, 321 Zhongshan Road, Nanjing, Jiangsu 210008 People’s Republic of China; 2 Joint Research Center for Bone and Joint Disease, Model Animal Research Center (MARC), Nanjing University, Nanjing, Jiangsu 210093 People’s Republic of China

**Keywords:** Prevalence, Patellofemoral pain, Knee pain, Association, Questionnaire survey

## Abstract

**Background:**

Previous studies that have described the prevalence of patellofemoral pain (PFP) have been limited to samples of military personnel or sporting populations, and convincing data in the general Chinese population are lacking. The present study defined the prevalence of PFP and knee pain in the general population of Chinese young adults and evaluated whether gender, age, or body mass index (BMI) were associated with PFP.

**Methods:**

An anonymous online questionnaire survey was open to the general public in China. A self-report questionnaire was used to specifically identify PFP. The population aged 18–40 years was enrolled in the study and completed the questionnaire. The prevalence of PFP and knee pain in the overall sample and in subgroups stratified by sex, age, and BMI was estimated. Logistic regression analysis was conducted to determine if there was a significant association between PFP and sex, age, or BMI.

**Results:**

A total of 1153 participants were enrolled in the study. The prevalence of PFP in the overall sample and among the male and female participants was 20.7, 20.3, and 21.2%, respectively. The prevalence of the knee pain in the overall sample and among the male and female participants was 35.6, 38.2, and 33.7%, respectively. The prevalence of PFP in the subgroups stratified by age and BMI did not differ significantly between the groups. Gender, age, and BMI did not have significant associations with the prevalence of PFP.

**Conclusion:**

PFP is common in the general Chinese population. Clinicians should direct more attention toward the early diagnosis of and interventions for PFP.

## Background

Patellofemoral pain (PFP) is a common lower extremity disorder in adolescents and physically active adults [1]. PFP is one of the most frequent causes of anterior knee pain and mainly affects young women [2, 3]. Patients with PFP often experience pain behind or around the patella, usually without any structural changes [4]. PFP leads to restrictions in activities of daily life; it may also be a risk factor of patellofemoral joint osteoarthritis (PFJOA) [5, 6].

Studying the epidemiology of PFP may be useful in preventing and controlling the disease. The prevalence of PFP is commonly reported as high, especially in young adolescents aged 12 to 17 years old [7]. A review study demonstrated that the prevalence of PFP may be up to 40% [8]. However, the majority of the studies regarding the prevalence of PFP were limited to samples of military personnel, sporting populations, or school children [8–11]. Thus far, few studies have examined data from general populations. Recently, the SNAPPS questionnaires have been developed to assess the prevalence of PFP within the general population. While they identified a prevalence rate of 22.7%, the study only included 111 patients [12]. There is still a need to evaluate the prevalence in a large-scale population-based study.

To our knowledge, convincing data in general Chinese populations is not yet available. Thus, the present study assessed the prevalence of PFP in the general population of Chinese young adults (aged 18–40 years) using a self-report questionnaire that was designed to identify individuals with PFP in the community.

## Methods

### Study design

This study was an anonymous online questionnaire survey of the general population of Chinese young adults. The Ethics Committee of Nanjing Drum Tower Hospital approved the survey. All subjects signed informed consents by marking a checkbox, and the information of the subjects was protected. A self-report questionnaire (SNAPPS-Survey Instrument for Natural History, Etiology, and Prevalence of Patellofemoral Pain Studies) [12] was transformed into an online format through a website (www.lediaocha.com). The questionnaire in this study was designed to be completed and spread through the social media platform WeChat, as almost everyone in China has WeChat on their mobile phone. Thus, people from various careers and different cities in China could be enrolled in this study.

### Participants

The sample was comprised of adults aged 18 to 40 years old who completed and submitted the questionnaire on the web platform. The participants included were engaged in a variety of occupations. Participants who were outside the age range or unable to complete the questionnaire were excluded from the study.

### Measures

The SNAPPS questionnaire used in this study was designed by Dey et al. in 2016 [12]. The questionnaire was designed to discriminate between the individuals in the community with and without PFP. The SNAPPS questionnaire was comprised of four sections. The first section identified individuals with knee pain based on one specific question. The second section addressed the clinical features of the knee problem. The third section encompassed difficulty or pain during specific activities that were commonly associated with a knee problem. The last section was designed to identify the location of the pain using a knee pain map. The designers conducted a comparative study including soft-tissue injury patients, PFP patients, and adults without knee problems [12]. The results showed that the measurement properties of the questionnaire were satisfactory based on the scoring, limited to sections 2 and 4, with a high sensitivity and specificity (both > 90%) [12]. The threshold score was set to ≥6 for PFP. Thus, our online SNAPPS questionnaire consisted of sections 1, 2, and 4 from the original questionnaire. In the first section, the participants provided their demographic information (sex, age, height, body weight) and answered the question “Have you had pain or problems in the last year in or around the knee?” If the answer was “no,” the questionnaire would be submitted, and the participants would be classified into the group of patients with no knee pain. If the answer was “yes,” the subjects would complete the remaining two sections (Table 1). All of the data were collected, and the scores on sections 2 and 4 were calculated. There were seven questions in section 2, and the participants could get “0” or “1” score for each question according to their answer. In section 2, the minimum score was 0 and the maximum score was 7 (Table 1). In section 4, a picture of a knee joint was shown with the medial, lateral and inferior part of the patella labeled. In total, six areas were labeled in both knees. The participants were asked to determine the number of areas in which they experienced pain in both knees. One point was awarded for each pain area selected. In section 4, the minimum score was 0 and the maximum score was 6 (Table 1). The final scores of the participants were shown by calculating the scores on sections 2 and 4. The participants with a total score < 6 were considered to have self-reported knee pain but not PFP. The participants with a total score ≥ 6 were considered to have PFP.

**Table 1:** Items and methods for scoring, according to SNAPPS

Items	Scoring of sections	
Patient characteristics		
Sex		
Age		
Height		
Body weight		
Section 1:	Answer	
	Yes	No
Have you had pain or problems in the last year in or around the knee?	Continue to finish sections 2 and 4	Submit
Section 2: Clinical features	Answers and scores	
	Yes	No
Did you have pain or problems in one or both of your knees?	1	0
Have you had surgery on your knee?	0	1
Have you ever had a knee cap that has gone out of joint (dislocated)?	0	1
Since the beginning of your knee problem, has your knee ever swelled up?	0	1
Have you had pain and discomfort for more than one month?	1	0
Thinking about your right (left) knee, what do you consider your main problem with your knee? Did you experience pain or discomfort?	1	0
Thinking about your right (left) knee, did your current knee problem come on?	1	0
In the presence of bilateral pain, a maximum score of 1 was given for each clinical feature	The minimum score was 0, and the maximum score was 7 in this section	
Section 4: Knee pain map	Scores	
The participants were asked to determine the number of areas in which they experience pain (total of six areas in both knees)	One area gets a score of 1	
	In this section, the minimum score was 0, and the maximum score was 6	

### Statistical analysis

Descriptive statistics were used to describe the characteristics of the participants. The prevalence of PFP and knee pain was calculated in the overall sample and in the male and female participants. The proportion of PFP participants in the knee pain population was also analyzed. The difference in the prevalence of PFP and knee pain among male and female groups was analyzed by a chi-square test. Logistic regression analysis was used to examine the association between each factor (gender, age, BMI) and the prevalence of PFP. Furthermore, the participants were categorized into small groups according to age (< 20, 20–30, and 31–40 years) as well as BMI (< 18.5, 18.5–24, and > 24). The above data were analyzed again in subgroups. *P* ≤ 0.05 was considered statistically significant. All of the statistical analyses were performed using SPSS 19.0 (SPSS Inc., Chicago, IL, USA).

## Results

A total of 1265 questionnaires were submitted. After excluding participants who were outside the age range or unable to complete the questionnaire, 1153 participants (676 females and 477 males) from different cities of China were enrolled in the present study. The mean age of the participants was 27.33 ± 4.38 years (Table 2). The mean BMI was 21.79 ± 2.92 (Table 2). However, no significant difference was observed in age between the male and female participants (27.39 ± 3.97 vs. 27.29 ± 4.66, *P* = 0.130). The BMI of the male subjects was significantly higher than that of their female counterparts (23.31 ± 2.92 vs. 20.72 ± 2.39, *P* < 0.001) (Table 2).

**Table 2:** Characteristics of the participants and the prevalence of PFP and knee pain

	Total	Men	Women	*P* value (Men vs Women)
Number of subjects	1153	477(41.4%)	676(58.6%)	–
Age (mean ± SD)	27.33 ± 4.38	27.39 ± 3.97	27.29 ± 4.66	0.130
BMI (mean ± SD)	21.79 ± 2.92	23.31 ± 2.92	20.72 ± 2.39	< 0.001
PFP	239 (20.7%)	97(20.3%)	142(21.2%)	0.711
Knee pain	410 (35.6%)	182(38.2%)	228(33.7%)	0.111
PFP/knee pain	239 (58.3%)	97(53.3%)	142(62.3%)	0.067

Next, the prevalence of PFP in the overall sample (239 of 1153, 20.7%) and in the male (97 of 477, 20.3%) and female (142 of 676, 21.2%) participants was calculated (Table 2). No significant difference was observed between the male and female groups (*P* = 0.711). Of all 1153 participants, 411 experienced knee pain, with a prevalence of knee pain of 35.6% (Table 2). More male participants experienced knee pain compared to their female counterparts (38.2 vs. 33.7%), although no significant difference was detected (*P* = 0.111). Furthermore, the proportion of participants with PFP with respect to knee pain was analyzed. The proportion of knee pain participants with PFP was 58.3% (239 of 410) in the total sample, 53.3% (97 0f 182) among the male participants, and 62.3% (142 of 228) among the female participants (Table 2). Logistic regression analysis was conducted to detect whether gender, age, and BMI were associated with PFP. The results showed that gender, age, and BMI were not related to the prevalence of PFP in the overall sample (Table 3).

**Table 3:** Logistic regression analysis to identify associated factors with the prevalence of PFP

Variable	With PFP	Without PFP	Univariate logistic regression		Multivariate logistic regression	
	*N* = 239	*N* = 914	OR(95%CI)	P	OR(95%CI)	P
Age^a^	27.23 ± 4.14	27.36 ± 4.45	0.993(0.962–1.026)	0.691	0.986(0.954–1.020)	0.416
BMI^b^	22.01 ± 2.95	21.73 ± 2.91	1.033(0.985–1.084)	0.180	1.051(0.995–1.111)	0.075
Gender^c^	97(40.6%)	380(41.6%)	1.042(0.780–1.392)	0.782	1.187(0.857–1.643)	0.302

Age: Mean ± SD

BMI: Mean ± SD

Gender: Male (percentage)

^a^ OR for age is for an increase of 1 year

^b^ OR for BMI is for a 1-unit increase

^c^ OR for gender uses male as the reference group

## Discussion

PFP mainly affects the young population. The prevalence of PFP in the general population still needs further exploration [12, 13]. To the best of our knowledge, this is the first study in China focusing on the prevalence of PFP and knee pain in the general population. The present study provided data on the general population of Chinese young adults and analyzed whether gender, age, or BMI were related to the prevalence of PFP.

The results showed that the overall prevalence of PFP in young Chinese individuals was 20.7%, whereas the prevalence in male and female subjects was 20.3 and 21.1%, respectively (Table 2). The prevalence of PFP that was reported by previous studies varied [2, 8, 12, 14–16]. Generally, the data indicated relatively lower prevalence in this study. Different personnel constitution of the sample structure and diagnostic methods may contribute to the differences in prevalence. The majority of previous studies were based on specific populations (e.g., military, students, and sporting personnel) that may perform higher levels of activity than the general population and may be of different ages compared to our study sample. On the other hand, unlike other studies, we used a self-report questionnaire to identify PFP, which may influence the results to some extent. The method we used may omit some cases or misclassify other knee pathologies as PFP.

In the present study, the prevalence of PFP in females was slightly higher than that in males (21.2 vs. 20.3%); however, no significant difference was detected. PFP is known to occur more frequently in females than in males [3, 17]. Several studies speculated that anatomical alignments and biomechanical factors might explain this phenomenon. Current prospective evidence showed that knee valgus displacement during landing could predict the risk of developing PFP [18]. Studies demonstrated that female athletes displayed increased knee abduction alignment compared to males during pivoting movement and drop landing [19, 20]. The prevalence of PFP was not significantly different between genders in the present study. Again, we supposed that different personnel-based sample structures and diagnostic methods might lead to the present results. This study also showed that PFP populations constituted more than half of the knee pain participants, which is sufficient to capture the attention of professionals to diagnose and treat PFP in clinical work.

A logistic regression analysis was conducted to identify if gender, age, and BMI were associated with the prevalence of PFP; however, such an association was not established (Table 3). Although the etiology of PFP has been shown to be multifactorial [21, 22], a consensus regarding the factors contributing to PFP is still lacking [23]. Several studies have been conducted to assess the factors associated with the prevalence of PFP. Some variables, such as BMI, height, mass, age, gender, hip weakness, and somatotype, have been examined; however, no significant associations have been found [2, 13, 24–28]. Higher-quality studies are needed to further explore the factors associated with the prevalence of PFP.

One feature of the present study was to use the social media platform WeChat to recruit participants and gather information. WeChat is very popular in China. Almost every adult has WeChat on their mobile phone. Thus, we can spread the questionnaire to various populations from different cities of China. The original SNAPPS designed by Dey et al. [12] contained four sections. However, their study showed that the measurement properties of the questionnaire were improved by omitting section 3 [12]. Additionally, too many questions in the questionnaire may have reduced the completion rate of our study. Based on these considerations, we removed section 3 in our online questionnaire.

The present study has some limitations. One of the main limitations is the lack of a standard definition of PFP. Both the clinical examination and self-reported questionnaire have shortcomings. The questionnaire is relatively suitable for a large-scale online-based survey. The SNAPPS used in this study has not been evaluated for diagnostic accuracy within a community setting. As mentioned above, some of the questions in the questionnaire may omit some cases or lead to misclassification of other knee pathologies as PFP. We defined the age range as 18–40 years, which may influence the results to a certain degree. The prevalence in older or younger populations may differ from that in the defined age range. Some juveniles may be restricted in their use of mobile phones in China, and adults older than 40 years old may get knee osteoarthritis. For this reason, only 18- to 40-year-old participants were enrolled in this study. The number of participants enrolled in the study was relatively small, which is another main limitation.

## Conclusion

The current community-based study is the first to evaluate the prevalence of PFP and knee pain in the general Chinese population. The study showed that the overall prevalence of PFP and knee pain in the sample was 20.7 and 35.6%, respectively. Gender, age, and BMI did not seem to be associated with the prevalence of PFP in the general Chinese population. There is still a need for an in-depth study with a large number of participants in China examining the prevalence of PFP and associated factors.

## Abbreviations

BMI: body mass index
PFJ: patellofemoral joint
PFP: prevalence of patellofemoral pain
SNAPPS: Survey instrument for Natural History, Etiology, and Prevalence of Patellofemoral Pain Studies
